# Diagnosis and treatment of severe tuberculosis complicated by ARDS and MODS in a young immunosuppressed host: a case report

**DOI:** 10.3389/fmed.2026.1846671

**Published:** 2026-06-30

**Authors:** Qiong Wang, Cheng Tang

**Affiliations:** Department of Respiratory and Critical Care Medicine, Zhuzhou Second Hospital, Zhuzhou, Hunan, China

**Keywords:** acute respiratory distress syndrome (ARDS), immunosuppressed hosts, multiple organ dysfunction syndrome (MODS), personalized therapy, severe tuberculosis

## Abstract

**Background:**

Severe tuberculosis (TB) presents with complex clinical manifestations and high mortality. Immunosuppressed hosts are at high risk for TB infection and prone to progress to severe disease.

**Case presentation:**

A young female patient was admitted to our Respiratory Intensive Care Unit with fever, cough, and progressive dyspnea. She had a history of nephrotic syndrome and was on long-term corticosteroids and immunosuppressive agents, but without TB screening at baseline. Following admission, she rapidly developed acute respiratory distress syndrome (ARDS), diffuse alveolar hemorrhage, and multiple organ dysfunction syndrome (MODS). While providing broad-spectrum anti-infective therapy, invasive mechanical ventilation, continuous renal replacement therapy, and supportive care to maintain vital signs, we achieved an early definitive diagnosis of disseminated tuberculosis through rapid sputum acid-fast bacilli staining, *Mycobacterium tuberculosis* nucleic acid testing, and metagenomic next-generation sequencing (mNGS). A multidisciplinary team collaborated to formulate an individualized anti-tuberculosis treatment plan, leading to a favorable clinical outcome.

**Conclusion:**

This case highlights the necessity of TB screening in immunosuppressed hosts, early recognition of severe TB, the importance of precise etiological diagnosis, and emphasizing the application of comprehensive treatment strategies in such patients.

## 1 Introduction

Tuberculosis remains a major global health threat. In 2023, there were an estimated 10.8 million new TB cases worldwide, with 741,000 in China (6.8% of global incidence), ranking third among high-burden countries (1). TB caused approximately 1.25 million deaths, reclaiming its position as the leading cause of death from a single infectious agent (1). Severe TB, often complicated by respiratory failure, septic shock, massive hemoptysis, or MODS, requires ICU care (2–4). Despite aggressive treatment, the mortality of severe TB remains as high as 22%–74% (3, 5, 6), posing significant challenges to TB control and the WHO’s “End TB” strategy targets.

Immunosuppressed individuals constitute a particularly vulnerable population to TB infection, including patients on long-term corticosteroid or other immunosuppressive therapies for conditions like malignancies, autoimmune diseases, nephrotic syndrome, etc. Their compromised cellular immunity, especially diminished CD4+ T-lymphocyte function, not only elevates the risk of primary TB infection or reactivation of latent disease but also predisposes them to rapid, disseminated disease (7, 8). In immunosuppressed hosts, the classic clinical and radiological signs of TB can be atypical or masked, leading to critical delays in diagnosis and treatment initiation.

Here, we present a challenging case of fulminant TB complicated by ARDS, diffuse alveolar hemorrhage, and MODS in a young woman with nephrotic syndrome on chronic immunosuppression. This case underscores the aggressive nature of TB in immunocompromised hosts and highlights the critical role of a rapid, comprehensive diagnostic approach centered on mNGS in enabling early, targeted therapy. We discuss the pathophysiological interplay between immunosuppression and disseminated TB, the strategic application of modern diagnostics, and the essentials of multidisciplinary management in the intensive care setting. This report aims to emphasize the necessity of heightened clinical suspicion for TB in similar high-risk patients and to illustrate how integrating advanced diagnostics can alter the clinical course and improve prognosis in severe, disseminated tuberculosis.

## 2 Case description

### 2.1 Admission assessment

A 33-year-old female was admitted to our Respiratory ICU on March 19, 2025. The patient developed a fever (Tmax 39 °C), dry cough, and exertional dyspnea after catching a cold on March 15, accompanied by fatigue, poor appetite, generalized myalgia, nausea, vomiting, and diarrhea with yellow watery stools. No hemoptysis or chest pain was reported. She sought treatment at a local hospital on March 16 and received anti-infective therapy. Her diarrhea, nausea, and vomiting got relieved, but fever, cough, and dyspnea persisted. A chest CT on March 18 showed bilateral interstitial pneumonia, and she was treated with meropenem and respiratory support therapy. However, her dyspnea progressively worsened. Subsequently, she was transferred to our emergency department on the same day, where she received meropenem (1 g q8h) plus trimethoprim-sulfamethoxazole (1.44 g q12h), albumin infusion, and diuretics. Her condition continued to deteriorate, with a PaO2/FiO2 ratio falling below 100 mmHg, necessitating endotracheal intubation and invasive mechanical ventilation. She was admitted to our department on March 19.

The patient was diagnosed with nephrotic syndrome in November 2023 due to generalized edema, oliguria, hypoalbuminemia, proteinuria, and hyperlipidemia, and improved after treatment. She was discharged on prednisone 50 mg daily, furosemide 20 mg twice daily, and rivaroxaban 10 mg daily for 3 months. On March 2, 2024, she was re-hospitalized at another hospital for recurrent generalized edema and oliguria. After improvement, she was discharged on prednisone 50 mg daily with regular outpatient follow-up and gradual dose reduction, torsemide 5 mg twice daily, and tacrolimus 1 mg twice daily. Until current admission, she was taking prednisone 25 mg daily. Personal, menstrual, and family history are unremarkable.

Laboratory tests revealed a white blood cell count of 8.9 × 10^9^/L, with neutrophils 8.0 × 10^9^/L, hemoglobin 78 g/L, platelets 57 × 10^9^/L, lymphocytes 0.3 × 10^9^/L. Arterial blood gas analysis showed the lowest oxygenation index (PaO2/FiO2 ratio) to be 66. Laboratory findings also indicated hepatic and renal impairment: total protein 34.6 g/L, albumin 19.2 g/L, total bilirubin 25.4 μmol/L, alanine aminotransferase 101 U/L, and aspartate aminotransferase 180 U/L. Renal function tests demonstrated urea 22.2 mmol/L, creatinine 140 μmol/L [estimated glomerular filtration rate (eGFR) 42.4 mL/min], and uric acid 663 μmol/L. Lactate dehydrogenase was 621 U/L, N-terminal pro-brain natriuretic peptide 3594 pg/mL, procalcitonin 1.784 ng/mL, and C-reactive protein 81.1 mg/L. T-cell subset counting: CD4+ T cells 152 cells/μL, CD8+ T cells 116 cells/μL, B cells 53 cells/μL, and NK cells: 56 cells/μL. The interferon-gamma release assay (IGRA) was positive. Coagulation profile showed prothrombin time 14.2 s, prothrombin activity 66%, activated partial thromboplastin time 58.7 s, fibrinogen 1.26 g/L, and D-dimer 3.82 mg/L. Tests for SARS-CoV-2 nucleic acid, influenza A/B antigen, erythrocyte sedimentation rate, rheumatology panel, antinuclear antibody profile, lupus panel, antineutrophil cytoplasmic antibody, vasculitis triad, thyroid function, and pre-transfusion four items were all negative. Electrocardiogram (ECG) revealed sinus tachycardia. Chest computed tomography (CT) performed at the emergency department on March 18 demonstrated interlobular septal thickening and diffuse small nodular, patchy, and ground-glass opacities in both lungs, with lower lobe predominance (Figure 1).

**FIGURE 1:**
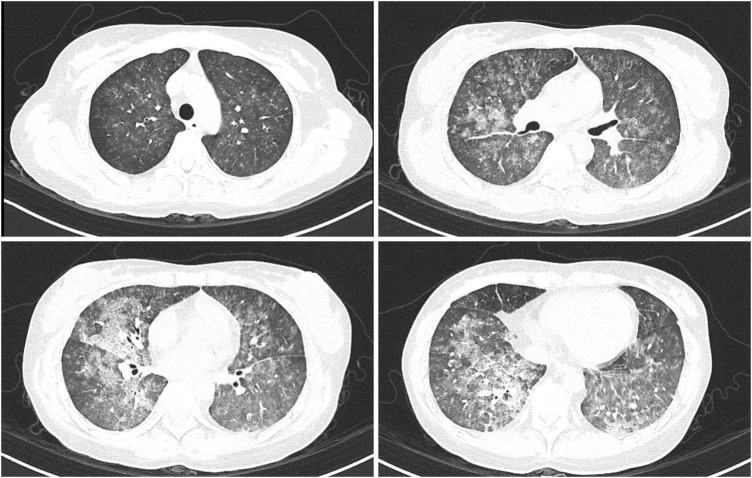
Chest computed tomography on March 18, 2025 revealed interlobular septal thickening and diffuse small nodular, patchy, and ground-glass opacities in both lungs, with lower lobe predominance.

### 2.2 Course of diagnosis and treatment

The patient, an immunosuppressed host due to nephrotic syndrome and long-term steroid use, presented with rapidly progressive bilateral diffuse lung lesions and ARDS. Given the possibility of pulmonary infection, anti-infective therapy covered common pathogens and opportunistic infections with meropenem, caspofungin, and trimethoprim-sulfamethoxazole was given, alongside nutritional support, albumin infusion, and diuresis. Upon ICU admission, we initially tapered the corticosteroid dose to prednisone 10 mg and discontinued tacrolimus. In the early hours of March 20, her respiratory failure worsened with oliguria and deteriorating oxygenation (lowest Peripheral Capillary Oxygen Saturation: 55%). Ventilator settings were adjusted (PEEP 14 cmH_2_O, FiO_2_ 100%). Emergency bedside bronchoscopy revealed patent airways with copious bloody serous fluid, which suggested diffuse alveolar hemorrhage (DAH); suction improved oxygenation slightly. Sputum culture, AFB stain, *Mycobacterium tuberculosis* DNA testing, anti-Glomerular Basement Membrane (GBM) antibody were performed. Immediate bedside continuous renal replacement therapy (CRRT) was initiated for pulmonary edema. Fresh frozen plasma and cryoprecipitate were transfused to improve coagulation.

On March 22, bronchoalveolar lavage fluid (BALF) GeneXpert MTB/RIF (Xpert) was positive for *Mycobacterium tuberculosis*, and the rifampicin resistance result was negative (rifampicin-susceptible). Sputum AFB stain was positive. Blood mNGS detected *Mycobacterium tuberculosis* (926 reads) exclusively; BALF mNGS also detected *Mycobacterium tuberculosis* (152 reads) with no other pathogens identified, confirming the diagnosis of tuberculosis. Anti-GBM antibody was negative. Thus, systemic prednisone treatment was stopped immediately to avoid exacerbating her immunosuppression. BALF Gomori methenamine silver stain was negative, which further excluded PJP; Thus, the dose of SMZ was adjusted to a prophylactic dose. Follow-up chest radiograph showed progression of diffuse pulmonary opacities in both lungs (Figures 2A, B). She was immediately transferred to a negative-pressure isolation room in ICU. A multidisciplinary discussion involving pulmonologists, respiratory therapist, TB specialists, nephrologists, and pharmacists led to an individualized treatment strategy. Meropenem and caspofungin were stopped. Considering liver injury, renal insufficiency, and ongoing CRRT, anti-tuberculosis therapy with isoniazid 0.3 g daily, ethambutol 0.75 g daily, and moxifloxacin 0.4 g daily, plus hepatoprotective agents, was initiated with monitoring of liver/kidney function and clinical status. Subsequently, we further screened for tuberculosis infection at other multiple sites. Urine AFB stain and Xpert were positive, stool Xpert was positive, and cerebrospinal fluid AFB stain and Xpert were negative. The positive urine AFB smear and Xpert for *Mycobacterium tuberculosis*, together with the positive stool Xpert result, confirmed the diagnosis of disseminated extrapulmonary tuberculosis involving the genitourinary tract and gastrointestinal tract. These findings provided strong evidence of hematogenous dissemination beyond the lungs, which is consistent with disseminated tuberculosis in an immunocompromised host. Following comprehensive treatment, her hemoglobin rose to 105 g/L, platelets normalized, coagulation markers improved, and ventilator FiO_2_ was weaned to 40%, PEEP to 8 cmH2O. Chest X-ray on March 24 and 26 showed that bilateral lung lesions significantly improved (Figures 2C, D).

**FIGURE 2:**
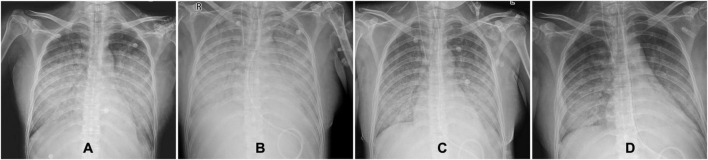
Serial Chest X-rays during hospitalization. **(A)** (March 19, 2025): Admission X-ray shows large, bilateral, roughly symmetrical patchy opacities with blurry borders, “butterfly” distribution, blurred costophrenic angles. **(B)** (March 22, 2025): Increased bilateral patchy opacities. **(C)** (March 24, 2025): Reduction in bilateral opacities, decreased density locally. **(D)** (March 26, 2025): Significant absorption of bilateral patchy opacities.

### 2.3 Follow-up

After confirmation of hematogenous disseminated tuberculosis and stabilization of her clinical condition, the patient was transferred to a specialized tuberculosis hospital on March 27 in accordance with China’s tuberculosis prevention and control regulations. She received anti-TB therapy included isoniazid 0.3 g daily, cycloserine 0.25 g twice daily, ethambutol 0.75 g daily, linezolid 600 mg every 12 h, and moxifloxacin 0.4 g daily. The endotracheal tube was removed on March 30, transitioning to High-Flow Nasal Cannula. She completed a full, standardized course of anti-TB treatment. A follow-up chest CT at 6 months post-discharge showed full absorption of lung lesions (Figure 3). The timeline of diagnosis, treatment, and outcomes for this patient was summarized in Figure 4.

**FIGURE 3:**
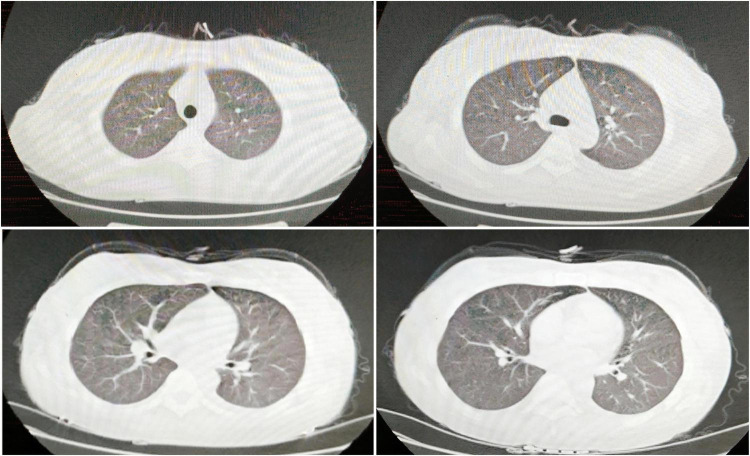
Follow-up chest computed tomography on October 13, 2025, showed no significant abnormality is observed in both lungs after standardized anti-TB treatment.

**FIGURE 4:**
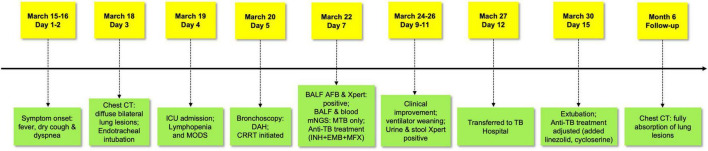
Timeline of diagnosis, treatment, and outcomes for this case. CRRT, continuous renal replacement therapy; BALF, bronchoalveolar lavage fluid; mNGS, metagenomic next-generation sequencing; MTB, *Mycobacterium tuberculosis*; AFB, acid-fast bacillus; Xpert, GeneXpert MTB/RIF; INH, isoniazid; EMB, ethambutol; MFX, moxifloxacin; DAH, diffuse alveolar hemorrhage; TB, tuberculosis; ICU, intensive care unit.

## 3 Discussion

### 3.1 Definition and risk factors of severe tuberculosis

Severe tuberculosis, characterized by atypical symptoms, rapid progression, and high mortality, poses a serious threat to global health security. Despite growing research on severe tuberculosis, significant challenges remain, including the absence of a uniform definition, standardized diagnostic criteria, and effective treatment protocols, all contributing to persistently poor outcomes (9). A meta-analysis on defining severe TB identified common criteria: involvement of >3 lung fields; patchy lesions with necrosis, cavities, or consolidation; severe complications; severe TB symptoms; or respiratory failure. The Chinese expert consensus categorizes severe TB into: Type I (severe parenchymal damage/hypoxemia), Type II (with severe complications), and Type III (critical, requiring ICU support for shock or MODS) (10). Type I and type II are more common, while type III is relatively rare.

Risk factors for severe TB include diabetes, advanced age, malnutrition, immunosuppression, renal dysfunction, and multidrug-resistant TB (MDR-TB). Diabetes correlates with latent TB infection and disease severity (11, 12). Immunosenescence increases the risk of severe TB in the elderly (13). Severe TB patients show elevated inflammatory monocytes and reduced lymphocytes (e.g., NK, γδ T cells), suggesting lymphopenia as a hallmark (14). Tuberculosis patients often present with concomitant malnutrition, which subsequently compromises their immune function. A multicenter, prospective cohort analysis indicated that malnutrition may lead to poor absorption of anti-tuberculosis drugs or increased drug toxicity, thereby affecting treatment efficacy and contributing to disease progression (15). Immunosuppression (HIV, autoimmune diseases, immunosuppressive therapy) is a well-established risk factor of severe TB (16, 17). Notably, long-term systemic corticosteroids at doses exceeding 15 mg/day of prednisone equivalents significantly increases the risk of developing tuberculosis (18). Besides, Chronic kidney disease affects immune response and drug dosing (19). MDR-TB (multidrug-resistant tuberculosis), with its prolonged treatment and complications, often leads to severe disease with low cure rates. Therefore, early identification and implementation of effective interventions are crucial for reducing the incidence of severe pulmonary tuberculosis and improving its prognosis.

This case involved a young female who was immunosuppressed due to long-term steroids and tacrolimus for nephrotic syndrome. Although chest CT performed before initiating immunosuppressive therapy for nephrotic syndrome ruled out active TB, latent TB infection (LTBI) screening was not performed, missing an opportunity for preventive therapy. Her significantly impaired cellular immunity, notably low CD4+ count, weakened immune surveillance against *M. tuberculosis* (20), leading to hematogenous dissemination and multi-organ involvement.

### 3.2 Diagnostic challenges

The diagnosis of disseminated tuberculosis in immunocompromised hosts remains notoriously challenging due to its non-specific clinical presentation, rapid progression and the frequent limited yield of conventional microbiological tests (21). In this case, DAH was confirmed by bronchoscopy and contributed significantly to the atypical radiological findings. The patient had multiple risk factors for DAH: severe thrombocytopenia (platelets 57 × 10^9^/L), coagulopathy (fibrinogen 1.26 g/L, elevated D-dimer 3.82 mg/L), and chronic high-dose corticosteroid use. These factors collectively predisposed to alveolar capillary injury and bleeding, which superimposed on the underlying disseminated tuberculosis. As a result, the chest CT showed diffuse small nodules, patchy opacities, ground-glass opacities, and lower lobe predominance – a pattern that deviates from the classic uniform 1–3 mm micronodules of miliary tuberculosis. This atypical appearance, caused by DAH, markedly increased the diagnostic difficulty. The case highlights that in severely immunocompromised patients, disseminated tuberculosis can present with non-classic clinical and radiological features. Therefore, even when chest CT does not show typical miliary nodules, tuberculosis should remain a routine consideration in the differential diagnosis of diffuse lung infiltrates, especially in the setting of unexplained alveolar hemorrhage or when risk factors for immunosuppression are present.

The cornerstone of managing severe TB is timely and accurate etiological diagnosis to guide precise anti-tuberculous therapy. Conventional diagnostic methods, including smear microscopy and culture, suffer from limited sensitivity and slow turnaround times. The emergence of molecular diagnostics, particularly mNGS, offers a paradigm shift. mNGS allows for rapid, unbiased pathogen detection directly from clinical samples and can be pivotal in identifying unsuspected infections in complex, critically ill patients (22). A recent study has demonstrated that mNGS of blood samples has excellent sensitivity for the diagnosis of disseminated tuberculosis, with higher detectability in patients with decreased CD4+ T cell counts—precisely the profile of our patient (22). Although severe TB was not initially suspected, rapid and comprehensive lower respiratory tract pathogen detection, especially mNGS, enabled timely etiological diagnosis in this patient.

In our case, the positive urine AFB smear and Xpert for *Mycobacterium tuberculosis*, together with the positive stool Xpert result, confirmed the diagnosis of disseminated (extrapulmonary) tuberculosis involving the genitourinary tract and gastrointestinal tract, even in the absence of typical urinary or gastrointestinal symptoms. These findings provided strong evidence of hematogenous dissemination beyond the lungs, which is consistent with disseminated tuberculosis in an immunocompromised host. Additionally, the presence of tuberculous bacilli in non-pulmonary specimens further underscores the utility of casting a broad diagnostic net beyond conventional respiratory samples when disseminated disease is suspected.

### 3.3 Pathophysiology of ARDS and MODS

The development of ARDS in this patient was most likely driven by a combination of overwhelming disseminated tuberculosis and severe immunosuppression. The massive bacillary load likely triggered a cytokine storm, contributing to widespread alveolar-capillary membrane injury, non-cardiogenic pulmonary edema (ARDS), and MODS. In tuberculosis-induced ARDS, elevated levels of pro-inflammatory cytokines such as CXCL8, TNF-α, and IL-6 have been implicated in the pathogenesis of alveolar injury. Specifically, CXCL8 levels in bronchoalveolar lavage fluid are significantly higher in patients with TB-induced ARDS compared to those with TB alone or ARDS alone, and these levels correlate with disease severity as measured by Murray scores (23). Furthermore, neutrophils have recently emerged as critical contributors to the deleterious immune response against M. tuberculosis, driving a hyperinflammatory process in the lungs during active TB that results in severe pulmonary damage (24). The combination of high bacterial load, impaired cellular immunity, and resultant neutrophil-mediated hyperinflammation likely explains the rapid progression to ARDS and MODS in this young patient.

### 3.4 Anti-tuberculosis treatment strategy

Anti-TB chemotherapy is the cornerstone, following principles of early, combined, regular, adequate, supervised, and individualized treatment. WHO and national guidelines recommend standard first-line regimens, with second-line drugs reserved for intolerance, complications, or drug resistance (25). Treating severe TB poses challenges, including altered drug absorption/bioavailability, dose adjustments for organ dysfunction, drug interactions, and adverse effects (26). The Chinese expert consensus on severe pulmonary tuberculosis emphasizes that anti-tuberculosis treatment follows the principles of early, combined, regular, adequate, full-course, and individualized therapy. Based on standardized first-line regimens, high-efficacy, low-toxicity second-line anti-TB drugs may be selected based on the patient’s specific condition (10). Therefore, patients with severe pulmonary tuberculosis often cannot be managed according to conventional recommended regimens. A precise, individualized treatment plan should be formulated based on factors such as therapeutic drug monitoring for anti-tuberculosis agents, management of complications, protection of organ function, and adjunctive therapies. Building upon a standardized first-line anti-tuberculosis regimen, the judicious inclusion of efficient and low-toxicity second-line anti-tuberculosis drugs, such as fluoroquinolones (27) and linezolid (28), should be considered according to the patient’s clinical characteristics, drug sensitivity, tolerability, and drug availability. Our patient’s ICU-phase regimen (isoniazid, ethambutol and moxifloxacin) was tailored to severe hepatic impairment (Child-Pugh B) and renal failure with CRRT. While this pragmatic choice averted immediate drug-induced toxicity and achieved a favorable short-term outcome, the post-ICU regimen did not include rifampicin because her liver function remained abnormal for a prolonged period, which deviates from the standard of care recommended by WHO guidelines. This case highlights that there remains substantial room for improvement in balancing between individualized adaptation and guideline-based standardization in the management of severe tuberculosis in complex, immunosuppressed hosts.

### 3.5 ICU management and clinical outcome

The management of severe tuberculosis with ARDS and MODS in the ICU setting requires a multidisciplinary approach integrating anti-tuberculosis chemotherapy, organ support, and careful monitoring of immunosuppressive therapy. This patient received CRRT for acute kidney injury, highlighting the complexity of drug dosing in this setting. The lack of therapeutic drug monitoring was a major limitation (see section “3.6 Limitations”). The favorable clinical outcome of this case underscores the importance of early etiological diagnosis, timely initiation of appropriate anti-TB therapy, and aggressive supportive care in critically ill patients with disseminated tuberculosis. However, the 6-month follow-up duration, while reassuring radiologically, does not capture the full treatment course outcome by WHO criteria, and longer-term monitoring for post-TB lung disease and potential relapse remains warranted.

### 3.6 Limitations

Several limitations of this case report should be acknowledged. First, as a single case, the findings and management decisions cannot be generalized to all patients with severe tuberculosis. Second, LTBI testing (e.g., interferon-gamma release assay) was not performed before the initiation of immunosuppressive therapy; therefore, it is not possible to definitively distinguish between reactivation of latent disease and primary progressive tuberculosis. Third, culture confirmation status and complete drug susceptibility testing were not available for all anti-tuberculosis drugs, which limits the ability to fully justify the selection of anti-tuberculosis regimen and to exclude drug resistance as a contributory factor. Fourth, although the patient had rifampicin-susceptible tuberculosis, rifampicin was omitted throughout the treatment course due to Child-Pugh class B hepatic impairment. This deviation from WHO standard of care represents an important limitation, even though the patient achieved a favorable clinical outcome. Fifth, therapeutic drug monitoring for anti-tuberculosis agents was not performed during the ICU stay, so we cannot confirm whether optimal drug exposures were achieved, particularly given the patient’s severe organ dysfunction and CRRT. Sixth, the follow-up duration is only 6 months; while remarkable radiological improvement was observed, this does not capture the full treatment outcome by WHO criteria (e.g., cure, treatment completion). Longer follow-up is required to assess the risk of relapse and post-tuberculosis lung disease. Despite these limitations, this case provides valuable lessons regarding the diagnostic and therapeutic challenges of disseminated tuberculosis in immunocompromised hosts.

## 4 Patient perspective

During the acute phase of critical illness, the patient was intubated, mechanically ventilated, and under deep sedation and analgesia; therefore, she could not provide real-time feedback. After extubation and transfer to the general ward, she recalled having only fragmented memories of her ICU stay, primarily of fear and confusion when she briefly regained consciousness. She expressed profound gratitude to the medical team for saving her life. Her family reported that they were initially terrified by her rapid deterioration but were reassured by the doctors’ explanations of the diagnosis of disseminated tuberculosis and the treatment plan. The patient and her family agreed to share this experience to help other patients facing similar life-threatening illnesses.

## 5 Conclusion

In summary, severe disseminated TB with complications poses diagnostic and therapeutic challenges, necessitating more effective individualized strategies. LTBI screening and preventive anti-TB treatment in immunosuppressed hosts are essential to mitigate severe TB risk. Rapid, precise etiological diagnosis using tools like mNGS and multidisciplinary management are key to improving outcomes in these critically ill patients.

## Data Availability

The original contributions presented in this study are included in this article/supplementary material, further inquiries can be directed to the corresponding author.
